# Supplementary material for: Dynalign II: common secondary structure prediction for RNA homologs with domain insertions
Source: Nucleic Acids Res. 2014 Nov 21;42(22):13939–48. doi: 10.1093/nar/gku1172 (PMC4267632; doi:10.1093/nar/gku1172)
Supplement: SUPPLEMENTARY DATA [file supp_gku1172_nar-02021-z-2014-File012.zip › manual/GUI/html/Secondary_Structure_Prediction_Algorithm.html]

RNAstructure GUI Help -- Secondary Structure Prediction Algorithm


|  |  |  |
| --- | --- | --- |
|  | RNAstructure GUI Help Secondary Structure Prediction Algorithm | - Contents - Index |
| Description of the Secondary Structure Prediction Algorithm  The "Fold RNA/DNA Single Strand" prediction of a secondary structure is based on Zuker's algorithm for free energy minimization, using the nearest neighbor parameters of Doug Turner and co-workers. A recursive algorithm is used that generates an optimal structure and a series of alternate structures.  These alternate structures are called suboptimal structures, defined as structures with free energy similar to the lowest free energy (optimal) structure. The number of suboptimal structures generated is controlled by two parameters: the maximum number of structures to be generated and % sort.  A third parameter, window size, may also be used. This controls how different the suboptimal structures must be from each other. A small window size allows generation of very similar structures, while a larger window size generates relatively more diverse structures.  For more details, see the description of the module "Fold."  Several alternative methods are also available for predicting secondary structures, including maximum expected accuracy (MEA) structure prediction, ProbKnot, which can predict pseudoknotted pairs, and stochastic sampling.   ---  **Accuracy**  A recent report (Mathews et al, see References) examined the accuracy of this method by comparing predictions to structures determined by comparative sequence analysis. When domains of less than 700 nucleotides were used, the algorithm was able to correctly predict 73% of base pairs from a database of 151,503 nucleotides containing small subunit rRNAs, large subunit rRNAs, group I introns, group II introns, RNase P RNA, SRP RNA, and tRNAs.  Accuracy can be dramatically improved by using constraints or restraints determined using experimental data. Alternatively, predicting a secondary structure common to multiple sequences improves the accuracy. For two sequences, this is done with Dynalign. For more than two sequences, this is done with Multilign.   ---  **Precision**  Within a small increment of free energy of the lowest structure, there can be many alternative structures for a helix appearing in the suboptimal structures. A dot plot can show how well defined a region of structure is. (See "Dot Plot.") Partition function predictions of base pairing probabilities provide specific details and structures can be color-annotated using pairing probabilities. | | |
| Visit The Mathews Lab RNAstructure Page for updates and latest information. | | |
